# Lifestyle Habits and Wellbeing Among Physicians in Pakistan: A Cross-Sectional Study

**DOI:** 10.7759/cureus.14875

**Published:** 2021-05-06

**Authors:** Anum S Siddiqui, Zubair Siddiqui, Ramsha Khulsai, Masood Jawaid

**Affiliations:** 1 Medical Affairs, PharmEvo Private Limited, Karachi, PAK; 2 Marketing, PharmEvo Private Limited, Karachi, PAK; 3 Surgery, Darul Sehat Hospital, Karachi, PAK

**Keywords:** lifestyle, wellbeing, physicians, wemwbs, physical activity, work life balance

## Abstract

Introduction

Physicians’ attitude towards a healthy lifestyle is important as it determines their extent of acceptance of these habits leading to overall well-being. Physicians with healthy lifestyle habits are more confident in advocating the same to their patients and their patients are keener in adopting lifestyle modifications. This study aimed to evaluate the lifestyle habits, wellbeing, and mental health of physicians in Pakistan.

Methods

A multi-center, cross-sectional survey was conducted with physicians across Pakistan from August to October 2020. A total of 1406 participants were gathered by the non-probability convenient sampling technique. Data were collected physically from the participants. A semi-structured, self-administered questionnaire comprising socio-demographic information, lifestyle habits, mental well-being, and well-being, in general, was filled. Mental wellbeing was assessed using the Warwick Edinburg Mental Wellbeing Scale (WEMWBS) comprising 14-positive items scored on a 1-5 point Likert scale where 1 corresponds to “none of the time” and 5 corresponds to “all of the time.” The score ranges from a minimum of 14 to a maximum of 70 points. Higher scores are associated with higher levels of mental wellbeing. Data were stored and analyzed using IBM-SPSS v.23 (IBM Corp., Armonk, NY).

Results

In this survey, there were 1284 (91.3%) males and 122 (8.7%) females with a mean age of 44.09 ± 11.18 years. More than half (n=768; 53.0%) of the physicians reported their general health as “good," 1045 (73%) were satisfied with their work-life balance, 206 (14%) had seen a physician for their health in the last six months, and 358 (25%) never had a routine medical checkup. When WEMWBS was applied, participants ≤40 years scored significantly higher than the older age group (p<0.01). Male physicians also scored significantly higher on WEMWBS (52.35 ± 8.78) as compared to their female counterparts (p<0.01). WEMWBS scores also varied significantly across various levels of expertise - with consultants scoring the highest (52.67 ± 9.02) and others scoring the lowest (48.63 ± 8.58; p=0.02). Physicians practicing in the public hospitals only (53.05 ± 9.02), scored higher on WEMWBS as compared to those in the private hospitals (51.28 ± 8.12) as well as those practicing in private clinics only (49.57 ± 8.82; p<0.01). Physicians who perceived their health as excellent scored highest on WEMWBS (53.55 ± 9.31), than those who considered their health good (51.68 ± 8.40), poor (50.71 ± 9.27), or fair (48.70 ± 8.15; p<0.01). The correlation analysis showed a significant negative correlation of WEMWBS scores with health in general (2.5% variation) and age (0.92% variation; p<0.01).

Conclusion

Physicians in our study were mostly satisfied with their general health and work-life balance. Nevertheless, their mental health well-being was not satisfactory, as assessed by WEMWBS. There is a dire need for lifestyle modifications among the medical practitioners who may improve their mental and physical well-being subsequently allowing them to cater to their patients more effectively. It is recommended that physician-patient-specific interventions should be developed to target the health status and mental well-being of a physician and to encourage the physicians especially the fresh medical graduates and young doctors to indulge in healthy activities.

## Introduction

The medical profession and its training have always been so tough that it inculcates a sense of independence, competitiveness, and resilience among physicians. Almost all physicians are exposed to everyday stressors, including prolonged and irregular working hours, the threat to workplace violence, disturbed sleep cycle, emotional exhaustion, and hence, more prone to stress-related disorders and other diseases [1]. One in every two physicians is seen to suffer from a major health issue by the time they turn 50 [2]. However, not all physicians are simply neglecting their health; there is a pattern of barriers associated with physicians’ health-seeking behavior as supported by research [1,3], with as many as 61% of physicians seen to be involved in self-diagnosis and self-treatment [1].

Over the past decade, there has been a change in this culture. Self-care has become a core competency by the Royal College of Physicians and Surgeons of Canada. Physicians are required to “demonstrate a commitment to physician health and sustainable practice [4].” Physicians are understanding that they are the main representatives of health and a healthy lifestyle. They play a leading role in advocating for a healthy lifestyle and physical activity for the masses [5]. Generally, people consider the physicians around them as role models of healthy behavior and lifestyle. Literature has shown evidence of physicians’ advice in helping quit unhealthy habits such as smoking and picking up good habits [6-8].

Scientific evidence has shown that physicians who practice healthy lifestyle habits are more confident in advocating the same to their patients and even their patients are keener in adopting lifestyle modifications in an attempt to prevent chronic diseases [9]. These physicians are more likely to communicate preventive lifestyle modifications to their patients. On the other hand, physicians who do not practice healthy lifestyles are less of an advocate of preventive measures and might not discuss them with their patients [5]. Physicians’ practices also determine their attitude towards advocacy of a healthy lifestyle. The physicians who take good care of their own physical and mental health counsel their patients more confidently and can relate to the barriers related to such lifestyle modifications. Physicians’ attitudes towards a healthy lifestyle are important as it determines their extent of acceptance of these habits leading to overall well-being. Most people refer to their physician as the main informant regarding a healthy lifestyle [10].

Among Pakistani physicians, a discrepancy in self-practice and patient-preaching has been identified. Where they advocated a healthy lifestyle, our physicians’ dietary habits, physical activity regime, and mental health were not seen to be up to the mark. A recent cross-sectional survey with more than 1000 Pakistani healthcare providers (HCPs) indicated that 76% did not exercise, 71% had >48 working hours per week, >50% slept for <7 hours daily, and their mental health averaged to a score of 48 (maximum 70) [11]. Comparable trends of unfavorable lifestyle habits are seen among physicians in other countries such as Bahrain and Saudi Arabia [5,12,13]. Although it is very important, the emphasis on physician health has been low in Pakistan as with many other countries [14]. Physicians practicing a healthy and balanced lifestyle will also be able to advise the same to their patients. This study aimed to evaluate the lifestyle habits, wellbeing, and mental health of physicians in Pakistan.

## Materials and methods

A multi-center, cross-sectional survey was conducted across various hospitals and private clinics in Pakistan from August to October 2020. The study was approved by the institutional review board (IRB # DSH/IRB/2020/0018). All participants were included after written informed consent.

The non-probability convenient sampling technique was adopted. The inclusion criteria consisted of physicians practicing in Pakistan as resident trainees, general practitioners (GP), and consultants, with a minimum clinical experience of three years, and those who willingly consented to participate in the specified duration of three months.

A semi-structured questionnaire was constructed which comprised of four sections. The first section consisted of socio-demographic information of the physicians including age, gender, professional status, institutional affiliation, marital status, body weight (kilograms), height (meters), chronic diseases, and family diseases. The second section consisted of lifestyle habits which included questions regarding food and drinks habits, sleep cycle, cigarette smoking, physical activity, and screen time. The third section comprised of well-being and included questions regarding self-perceived general health (participants' own view about their health as excellent, good, fair, poor), regular medical checkups, and work-life balance. The fourth section comprised mental wellbeing. It was assessed using Warwick Edinburg Mental Wellbeing Scale (WEMWBS). It is a reliable and validated tool with internal consistency and a reliability coefficient (Cronbach's alpha) of 0.87 [15]. It comprises 14-positive items and is scored on a 1-5 point Likert scale where 1 corresponds to “none of the time” and 5 corresponds to “all of the time.” All questions are equally weighted. Scores can range from a minimum of 14 to a maximum of 70 points. Higher scores are associated with higher levels of mental wellbeing [15].

Data were stored and analyzed using IBM-SPSS version 23.0 (IBM Corp., Armonk, NY). Cronbach alpha was used to check the reliability of the WEMWBS scale. Mean and standard deviation (SD) were given for continuous items. Frequency and percentages were calculated for categorical items. One-way analysis of variance (ANOVA) was used to compare the mean WEMWBS scores with marital status, profession, institutional affiliation, and health in general whereas an independent sample t-test was used to compare the mean WEMWBS with respect to age group and gender. Pearson Correlation analysis was performed to study the relationship of WEMWBS scores with age, health in general, and body mass index (BMI). P-values under 0.05 were considered significant.

## Results

There were 1,406 physicians included from various parts of the country for this survey. Most of them (n=1284; 91.3%) were males and 122 (8.7%) were females. Their mean age was 44.09 ± 11.18 years and their mean BMI was 25.09 ± 5.60 kg/m^2^. The majority (n=501; 43.1%) were general practitioners and the rest were consultants 418 (35.9%) and residents 173 (14.9%). Most of the study sample (n=1153; 83.4%) were married; 188 (13.6%) were single, and 42 (3.0%) were separated/divorced. There were 619 (46.4%) physicians who were practicing in public hospitals, 385 (28.5%) were in private hospitals, and 330 (24.7%) only practiced outpatient in the private sector. There were 664 (45.1%), hypertensive physicians, with 731 (49.7%) having a family history of hypertension; 520 (35.4%) diabetic physicians with 574 (39.0%) having a family history of diabetes; and 495 (33.7%) hypercholesterolemic with 138 (9.4%) having a family history of hypercholesterolemia. Most of them were prescribed angiotensin receptor blockers (n=354; 24.1%) for hypertension, metformin (n=268; 18.2%) for diabetes mellitus, and rosuvastatin (n=288; 19.6%) for hypercholesterolemia. Only 89 (6.1%) physicians were taking insulin for the treatment of diabetes mellitus.

There were 768 (53.0%) physicians who reported their general health as “good.” Only 206 (14.6%) had seen an HCP for their health in the last six months while 358 (24.7%) never went for a routine medical checkup. Around 72.8% (n=1045) of physicians were satisfied with their work-life balance and 16.4% (n=237) reported that if given the chance, they will choose another profession for themselves (Table 1).

**Table 1:** Self-reported well-being status of the physicians

Variables	Frequency (%)
Regular meals	
Breakfast	1184 (78.3%)
Lunch	981 (64.9%)
Dinner	1093 (72.3%)
Usual frequency of types of food	
Fresh fruits	
Do not eat	46 (3.0%)
1–2 times/week	381 (25.2%)
3–5 times/week	703 (46.5%)
6–7 times/week	321 (21.2%)
Fresh vegetables	
Do not eat	65 (4.3%)
1–2 times/week	437 (28.9%)
3–5 times/week	671 (44.4%)
6–7 times/week	271 (17.9%)
Chicken	
Do not eat	208 (13.8%)
1–2 times/week	779 (51.5%)
3–5 times/week	362 (23.9%)
6–7 times/week	72 (4.8%)
Fish	
Do not eat	149 (9.9%)
1–2 times/week	864 (57.1%)
3–5 times/week	356 (23.5%)
6–7 times/week	53 (3.5%)
Red meat	
Do not eat	281 (18.6%)
1–2 times/week	764 (50.5%)
3–5 times/week	341 (22.6%)
6–7 times/week	41 (2.7%)
Junk food	
Do not take	386 (26.5%)
Daily	96 (6.6%)
Once weekly	321 (22.0%)
Weekly	287 (19.7%)
Monthly	366 (25.1%)
Frizzy drinks	
Do not take	456 (31.5%)
Daily	159 (11.0%)
Once weekly	314 (21.7%)
Weekly	283 (19.5%)
Monthly	237 (16.4%)
Caffeinated beverages per day	
Do not take	585 (41.3%)
Once	507 (35.8%)
Twice	234 (16.5%)
Three times or more	92 (6.5%)
Mean glasses of water per day	8.56 ± 3.87
Physical activity in a week	
Yes	800 (56.3%)
Mean minutes/day physical activity	32.58 ± 18.9
Mean days/week physical activity	5.92 ± 9.80
Type of physical activity	
Lift weights	75 (6.6%)
Run	244 (21.6%)
Walk	717 (63.5%)
Swim	69 (6.1%)
Aerobics	24 (2.1%)
Others (cricket, football, badminton, yoga, etc.)	55 (3.7%)
Elevator or stairs use usually in multi-story buildings	
Elevators	209 (14.9%)
Stairs	536 (38.2%)
Both	658 (46.9%)
Mean hours of sleep per day	7.64 ± 1.39
Smoking status	
Current smoker	190 (13.1%)
Ex-smoker	220 (15.2%)
Never smoked	1040 (71.7%)
Mean hours in daily clinical practice	
Institution	6.25 ± 2.06
Private	5.30 ± 2.73
Mean average daily screen time	
TV/DVD	1.98 ± 2.95
Laptop/computer	2.46 ± 3.74
Mobile	3.75 ± 4.66

Table 2 summarizes the lifestyle habits of our sample physicians. These include their eating habits with most of the participants eating all three meals of the day. When inquired about fresh fruit intake, 46.5% (n=703) were found to be taking it 3-5 times/week, and for fresh vegetables, it was 3-5 times/week as well (n=671; 44.4%). Chicken and fish intake was 1-2 times/week in 51.5% (n=779) and 57.1% (n=864), respectively. Similarly, they reported eating red meat 1-2 times/week (n=764; 50.5%) and junk food monthly (n=366; 25.1%) or once weekly (321, 22.0%). Most of the study participants were not used to drinking fizzy (n=456; 31.5%) or caffeinated drinks (n=585; 41.3%). A good proportion (n=800; 56.3%) of physicians had a habit of doing physical activities at 32.58 ± 18.9 minutes/day and 5.92 ± 9.80 days/week like walking, running, and weight lifting. Their mean duration of sleep was 7.64 ± 1.39 hours/day while that of clinical practice was 6.25 ± 2.06 hours institutionally and 5.30 ± 2.73 in private.

**Table 2:** Lifestyle habits of the physicians

Variables	Frequency (%)
Regular meals	
Breakfast	1184 (78.3%)
Lunch	981 (64.9%)
Dinner	1093 (72.3%)
Usual frequency of types of food	
Fresh fruits	
Do not eat	46 (3.0%)
1–2 times/week	381 (25.2%)
3–5 times/week	703 (46.5%)
6–7 times/week	321 (21.2%)
Fresh vegetables	
Do not eat	65 (4.3%)
1–2 times/week	437 (28.9%)
3–5 times/week	671 (44.4%)
6–7 times/week	271 (17.9%)
Chicken	
Do not eat	208 (13.8%)
1–2 times/week	779 (51.5%)
3–5 times/week	362 (23.9%)
6–7 times/week	72 (4.8%)
Fish	
Do not eat	149 (9.9%)
1–2 times/week	864 (57.1%)
3–5 times/week	356 (23.5%)
6–7 times/week	53 (3.5%)
Red meat	
Do not eat	281 (18.6%)
1–2 times/week	764 (50.5%)
3–5 times/week	341 (22.6%)
6–7 times/week	41 (2.7%)
Junk food	
Do not take	386 (26.5%)
Daily	96 (6.6%)
Once weekly	321 (22.0%)
Weekly	287 (19.7%)
Monthly	366 (25.1%)
Frizzy drinks	
Do not take	456 (31.5%)
Daily	159 (11.0%)
Once weekly	314 (21.7%)
Weekly	283 (19.5%)
Monthly	237 (16.4%)
Caffeinated beverages per day	
Do not take	585 (41.3%)
Once	507 (35.8%)
Twice	234 (16.5%)
3 times or more	92 (6.5%)
Mean glasses of water per day	8.56 ± 3.87
Physical activity in a week	
Yes	800 (56.3%)
Mean minutes/day physical activity	32.58 ± 18.9
Mean days/week physical activity	5.92 ± 9.80
Type of physical activity	
Lift weights	75 (6.6%)
Run	244 (21.6%)
Walk	717 (63.5%)
Swim	69 (6.1%)
Aerobics	24 (2.1%)
Others (cricket, football, badminton, yoga, etc.)	55 (3.7%)
Elevator or stairs use usually in multi-story buildings	
Elevators	209 (14.9%)
Stairs	536 (38.2%)
Both	658 (46.9%)
Mean hours of sleep per day	7.64 ± 1.39
Smoking status	
Current smoker	190 (13.1%)
Ex-smoker	220 (15.2%)
Never smoked	1040 (71.7%)
Mean hours in daily clinical practice	
Institution	6.25 ± 2.06
Private	5.30 ± 2.73
Mean average daily screen time	
TV/DVD	1.98 ± 2.95
Laptop/computer	2.46 ± 3.74
Mobile	3.75 ± 4.66

Table 3 shows the descriptive statistics on WEMWBS items, where 899 samples fully completed the well-being questionnaire. The reliability of 14 items for the WEMWBS scale was observed at 0.87 using Cronbach alpha. The overall mean score for WEMWBS was 52.08 ± 8.26. The comparison of WEMWBS scores across socio-demographic factors showed that 46.3% of the sample was of ≤40 years with a mean of 52.97 ± 8.84 on WEMWBS which was slightly higher than the older age group and the differences were statistically significant with p<0.01. Male physicians also scored significantly higher on WEMWBS (52.35 ± 8.78) as compared to their female counterparts (p<0.01). WEMWBS scores also varied significantly across various levels of expertise - with consultants scoring the highest (52.67 ± 9.02) and others scoring the lowest (48.63 ± 8.58; p=0.02). Physicians practicing in the public hospitals only (53.05 ± 9.02), scored higher on WEMWBS as compared to those in the private hospitals (51.28 ± 8.12) as well as those practicing in private clinics only (49.57 ± 8.82; p<0.01). Physicians who perceived their health as excellent scored highest on WEMWBS (53.55 ± 9.31), than those who considered their health good (51.68 ± 8.40), poor (50.71 ± 9.27), or fair (48.70 ± 8.15). The differences were statistically significant (p<0.01).

**Table 3:** Descriptive statistics on WEMWBS items (n=899)

Items	Mean	SD
I have been feeling optimistic about the future	3.47	1.164
I have been feeling useful	3.69	1.008
I have been feeling relaxed	3.68	0.990
I have been feeling interested in other people	3.44	1.150
I have had the energy to spare	3.53	1.054
I have been dealing with problems well	3.80	0.993
I have been thinking clearly	3.88	1.019
I have been feeling good about myself	3.92	0.982
I have been feeling close to other people	3.55	1.084
I have been feeling confident	3.98	0.979
I have been able to make up my mind about things	3.85	0.983
I have been feeling loved	3.68	1.091
I have been interested in new things	3.90	1.000
I have been feeling cheerful	3.73	1.022
Reliability coefficient Cronbach’s α	14-items	0.87

The correlation analysis for WEBWBS scores with age, health in general, and BMI showed a significant negative correlation of WEMWBS scores with health in general and age. A 2.5% variation in WEMWBS explained by health in general and a 0.92% variation by age. These were considered statistically significant (p<0.01). However, there was no significant correlation observed between WEMWBS and BMI.

## Discussion

Stressful environments such as those of a patient-loaded hospital can lead to delayed or even misdiagnosis of diseases. Furthermore, due to long and exhausting work hours, physicians tend to overlook their own symptoms, hence, do not get a professional assessment for their conditions. Similarly, due to exhausting work hours, many physicians do not indulge in physical exercise or stick to their dietary regimes. They do not find the physical or mental strength to take care of themselves after working endless hours each day. This directly affects the quality of healthcare they provide to their patients [16,17]. The current study explored the lifestyle habits, wellbeing, and mental health of Pakistani physicians. This can be a step forward to develop certain national-level policies for improving the work efficiency of these HCPs.

The majority of the participants were satisfied with their health status; however, they scored poorly when it came to their mental health. Moreover, gender discrepancy was prominently noticed in this mental well-being assessment by the WEMWBS with males scoring significantly higher (p<0.01). This inequality of male to female physician ratio has been observed, globally [18]. Our findings were in accordance with a 2017 study by Khattak et al., where a physician’s physical and mental health were assessed, concluding that female physicians struggled with maintaining healthy activities affecting their overall health and well-being [19]. The authors recommend that to ensure physicians' well-being, novice interventional programs could be arranged that target this neglected area.

Duty hours of doctors and the need to restrict them have been long debated in the medical literature. For instance, a study from Indonesia, exploring the prevalence and risk factors of gastroesophageal reflux disease (GERD) in medical practitioners reported long working hours to be a contributing factor with doctors in Indonesia working 51-67 hours per week on average. They are under extremely stressful circumstances and the majority of the doctors are under constant worry about their patients [20]. Countries like the United States and New Zealand have managed to restrict doctor duty hours to 16-24 hours; however, in Pakistan, the situation is still unregulated [17]. Pakistani doctors are still working for as many as 74-92 hours weekly and are shown to exhibit high stress and low job satisfaction [21]. In the current study, the majority of the participants claimed that the most physical activity they get is by walking which includes walking from one department to the other. Only about one-fourth population confessed that they indulged in smoking which could be an understatement as many young residents or physicians tend to hide their habit of smoking. These unhealthy activities lead to poor health status among physicians [20,22,23].

One striking finding in our study was how one-fourth of the participants never visited a general health practitioner for a routine health examination. Further review of the literature divulged similar findings. In 2012, a review was conducted on preventive healthcare and lifestyle behaviors of medical practitioners towards their health [23]. It was concluded that there is a culture that prevents doctors from seeing other healthcare professionals when they feel sick. Doctors are often portrayed as heroes who do not need to seek any medical attention and their sole purpose is to serve patients. Asking for help is looked upon as weakness and those who do ask for help are judged. However, physicians who are physically and mentally fit and consider their health as a priority tend to provide better healthcare to their patients [22,23].

Wiskar in a review discussed the option of counseling physicians who indulge in detrimental habits and ignore their health, both mental and physical to fulfill their obligations as a physician [23]. Many doctors avoid going to a professional healthcare practitioner even when they are fully aware of their symptoms because there is a widely popular culture of associating a physician’s own mental and physical health with his/her medical competence. This is why many physicians tend to hide their conditions to avoid being judged by their fellow practitioners [22].

Not only does the health of a physician directly impact the delivery of the healthcare services to their patients but also affects the way they counsel their patients. In a Canadian study, it was found that physicians who were non-smokers were able to counsel their patients about smoking hazards actively and more confidently [24].

A study in 2014 proposed the implementation of a revised system in the medical field to allow the physician to take care of themselves [25]. The authors focus on work-life balance for not only physicians but also for other personnel of the hospital staff since these people are prone to suffer from work-related burnout and mental exhaustion. The study highlighted that it is important for the physician to be physically and mentally fit to provide better healthcare to their patients. The significance of work-life balance among healthcare professionals is highlighted in the current study as over one-fourth of the physicians were either not satisfied with their work-life balance or were unsure about it.

Another study reported that around 46% of doctors in the United States have felt frustrated with the system at some point in their careers [26]. In another study, 73% of the internists and 68% of the physicians claimed that they would reconsider their field of the profession if it was an option [27]. Contrary to that, our findings reported that only about one-sixth of the physicians wished to change their fields. Many of these physicians suffered from work-related burnout and reported a lack of interest in the activities of daily life, indulged in substance abuse, and had suicidal thoughts. These issues are preventable with certain modifications such as reasonable work hours, increased salary, increased leisure time, and indulging in self-care activities.

A study was conducted on the lifestyle of physicians highlighting the consumption of an unbalanced diet by the physicians [23]. Most of the physicians admitted to not having proper meals or a stable sleeping schedule due to their demanding jobs. The physicians were badly affected due to poor nutrition which subsequently affected their behavior and decision-making capabilities. The majority of the participants in our study consumed junk food (such as sweets, sweetened beverages, fast foods, and salty snacks) and carbonated drinks. Poor diet is associated with high-stress levels and poor cognitive functions [28]. 

Despite the many apparent strengths of the current study, there were some limitations. First, the participants were selected using the non-probability convenience sampling technique which may have raised the selection bias hence, limiting the interpretation of the findings. Second, the majority of the participants were male and married. Therefore, it impacts the generalizability of the data and limits the implication of the findings to the entire country.

## Conclusions

Physicians in our study were mostly satisfied with their general health and work-life balance. Nevertheless, their mental health well-being was not satisfactory, as assessed by WEMWBS. There is a dire need for lifestyle modifications among the medical practitioners who may improve their mental and physical well-being subsequently allowing them to cater to their patients more effectively. It is recommended that physician-patient-specific interventions should be developed to target the health status and mental well-being of a physician and to encourage the physicians especially the fresh medical graduates and young doctors to indulge in healthy activities.
